# Analysis of Barriers to Public and Private Cardiac Rehabilitation Programs in Patients with Low and High Adherence

**DOI:** 10.21470/1678-9741-2021-0436

**Published:** 2023

**Authors:** Lorena Altafin Santos, Dyovana Gomes Pinheiro, Jéssica Malek da Silva, Giovanna Lombardi Bonini Borges, Paula Fernanda da Silva, Ana Laura Ricci-Vitor, Luiz Carlos Marques Vanderlei

**Affiliations:** 1 Physiotherapy Department, Faculdade de Ciências e Tecnologia, Universidade Estadual Paulista “Júlio de Mesquita Filho” (UNESP), Presidente Prudente, São Paulo, Brazil

**Keywords:** Cardiac Rehabilitation, Health Services Accessibility, Attitude of Health Personnel, Choice Behavior, Patient Preference, Cardiovascular Diseases.

## Abstract

**Introduction:**

Participants in cardiac rehabilitation programs have low adherence to their sessions, which makes extremely important to recognize the barriers that cause non-adherence, identifying whether the type of service and level of adherence influence these barriers.

**Methods:**

This is a cross-sectional observational study, in which 220 individuals (66.80±11.59 years) of both genders who are members of public and private exercise-based cardiac rehabilitation programs participated. The volunteers were divided according to the level of adherence, considering patients with low adherence (PLA) those with < 70% of attendance and high adherence (PHA) those with > 70%. Then, initial evaluation, Cardiac Rehabilitation Barriers Scale, analysis of socioeconomic level, Hospital Anxiety and Depression Scale, and Mini-Mental State Examination were applied.

**Results:**

Higher total barriers were found in PLA in the public service compared to PHA in the private service (P=0.023). In the subscale “perceived need”, PHA in the public service showed higher values than PLA and PHA in the private service (P≤0.001). The “access” barrier was higher for PHA in the public service when compared to PHA in the private service (P=0.024). PHA in the public service exhibited a higher barrier regarding questions about distance, transportation problems, cost, and time constraints.

**Conclusion:**

The public program presents higher barriers in the questions and categories compared to the private program, mainly the PHA. Furthermore, there are differences in the profile of the participants regarding socioeconomic and anxiety levels, treatment time, ethnicity, and city where they live.

## INTRODUCTION

Although the benefits of cardiac rehabilitation (CR) programs^[^1^-^3^]^ are clear, participation and adherence to these programs are major challenges that need to be studied in Brazil and worldwide^[^2^,^4^-^6^]^. To obtain the beneficial effects of these programs, a minimum attendance of 70% to sessions is required^[^7^]^, which is a great challenge because the participants present several barriers to adherence such as travel, work conflicts, program costs, distance, personal problems, family responsibilities, comorbidities, access, and perceived needs^[^2^,^4^-^6^]^. Also, the type of financing of the CR programs, public or private health system, seems to influence these barriers^[^4^,^5^,^8^]^.

Studies related to barriers to CR do not make it clear whether they are different when considering the attendance of 70% as the cutoff point or when considering the different means of program financing. To answer these questions, it is intended, in this study, to compare the barriers presented by individuals with low or high adherence to CR and to compare these barriers between the public and private programs. As a secondary objective, we aim to compare the profile of the participants, allowing a better understanding of these patients’ adherence to these programs. We hypothesize that participants with low adherence in the public service present higher barriers.

## METHODS

### Study Design

This is a cross-sectional observational study, prepared according to the recommendations of the STrengthening the Reporting of OBservational studies in Epidemiology (or STROBE)^[^9^]^. The study started with a meeting with each patient, from May/2017 to June/2019, in which an initial evaluation was carried out to identify, characterize, and classify patients into two groups (high or low adherence), and classify them according to participation in the public or private program.

Then, the participants answered a questionnaire to investigate the barriers to CR^[^10^]^ and, later, they answered three questionnaires that investigated socioeconomic, anxiety, and depression levels and cognitive capacity. After the initial evaluation and application of the questionnaires, the profile and barriers to rehabilitation were compared considering the level of adherence and the nature of the program (public or private).

### Participants and Scenario

Participants were recruited for convenience in two exercise-based CR programs offered in the city of Presidente Prudente (São Paulo, Brazil), being one private and the other public. About CR programs, the public service is financed by the Brazilian Unified Health System, which serves about 18 patients per session and the duration of treatment is indefinite. The private sector, on the other hand, is financed by the patient or by health insurance, and 12 patients are treated per session. Most patients in the private program have medical insurance, which is paid monthly and covers 36 sessions. For those who do not have health insurance, the cost of the treatment is approximately 76 United States dollars per month. Both programs consist of the following phases: rest, warm-up, resistance, and relaxation. In the private program, the resistance phase differs from the public program, because in addition to the treadmill and bicycle activities, resistance exercise is performed.

As eligibility criteria, the list of all participants who attended the CR program was initially obtained and all patients over 18 years of age, diagnosed with cardiovascular disease or presence of risk factors and comorbidities that do not prevent the performance of CR, regardless of gender and attendance percentage, and who attended CR for at least three months were included. Those who were not found after three visits for evaluation were excluded.

After evaluating the eligibility criteria and the initial invitation, the participants were previously informed about the procedures and aims of this study and after agreeing to participate, they signed a written consent form. The procedures of the study were approved by the Committee for Ethics and Research of the Faculdade de Ciências e Tecnologia, Universidade Estadual Paulista “Júlio de Mesquita Filho” (CAAE: 88504718.0.0000.5402).

### Initial Evaluation

In the initial evaluation, the following information was obtained: age, anthropometric data (mass and height for subsequent calculation of body mass index [BMI]), gender, ethnicity, treatment time, main diagnosis, presence of risk factors, current occupation, city of residence, and educational level.

### Evaluated Outcomes

As outcomes, adherence, barriers presented by patients, socioeconomic, anxiety, and depression levels, and cognitive status were evaluated. Adherence was assessed by the attendance obtained over 36 sessions recorded in the patient charts. After adherence analysis, patients from both services were classified into two subgroups: patients with low adherence (PLA), with adherence values that corresponded to a session attendance < 70%, and another of patients with high adherence (PHA), showing attendance > 70%^[^7^]^.

The evaluation of the barriers was carried out through the Cardiac Rehabilitation Barriers Scale (CRBS)^[^10^]^, which has a general score or can be divided into five subscales: comorbidities/functional status, perceived need, personal/family issues, travel/work conflicts, and access^[^10^]^.

The socioeconomic level was assessed by the questionnaire of the Associação Brasileira de Empresas de Pesquisa^[^11^]^, which estimates the economic power of the individual and includes questions about educational level, family income, possession of items, and public services offered at the residence^[^12^]^. From the score obtained in the questionnaire, patients were classified in classes A to E.

To quantify the level of anxiety and depression, the Hospital Anxiety and Depression Scale was applied^[^13^]^. Cognitive status was analyzed using the Mini-Mental State Examination, and the presence or absence of cognitive deficit was adjusted based on educational level^[^14^]^.

### Statistical Analysis

Descriptive statistics were used to characterize the population, and the values were presented as mean and standard deviation or in absolute and percentage numbers. The evaluated outcomes were presented as mean, standard deviation, median, and lower and upper limit of the 95% confidence interval.

To compare the quantitative variables between the four groups, the Kolmogorov-Smirnov test was initially performed to test the normality of the data, followed by the Kruskal-Wallis test with Dunn’s post-test. The categorical variables were compared using the Chi-square test. Analyzes were performed using IBM Corp. Released 2013, IBM SPSS Statistics for Windows, version 22.0, Armonk, NY: IBM Corp. with statistical significance fixed at 5%.

## RESULTS

**Fig. 1:**
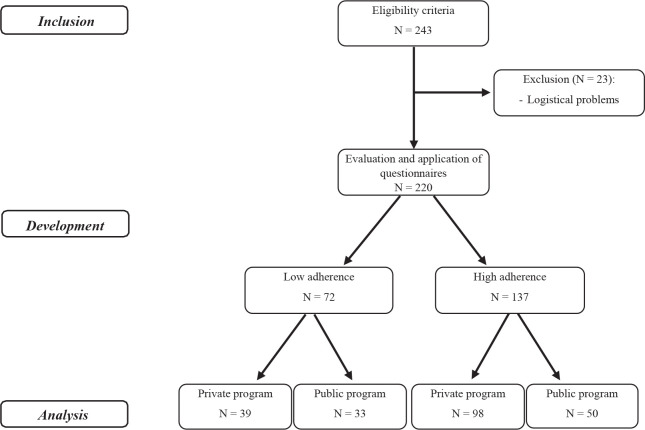
Patients’ distribution flowchart.

Two hundred forty-three individuals were considered eligible to participate in the study, among which 23 were not found after three visits for evaluation and were excluded. Of the 220 participants evaluated, 72 were allocated to the PLA group, 39 from the private program and 33 from the public program, and 137 were allocated to the PHA group, of which 98 belonged to the private program and 50 to the public program (Figure 1).

The characteristics of the participants considering the level of adherence (low or high) and the program (public or private) can be observed in Tables 1 and 2. Differences were found in the prevalence of black patients (*P*=0.026), being higher in PLA in the public service, and treatment time (*P*=0.002) was higher in PHA in the public sector compared to PLA and PHA in the private service. Considering the city of residence, the majority of PLA in the public and PHA in the private service resides in X (*P*=0.049). Also, higher levels of anxiety were found in PLA in the public service compared to PLA and PHA in the private sector (*P*=0.001), and about socioeconomic level, more PHA in the private service are classified at level A, while more PLA in the public service are classified at level C1 (*P*<0.001).

**Table 1:** Characterization of patients concerning gender, age, BMI, ethnicity, treatment time, medication use, occupation, and city of residence according to the type of program and adherence.

Characterization	Low Adherence	Low Adherence	High Adherence	High Adherence	*P*-value
	Public Program (n = 33)	Private Program (n = 39)	Public Program (n = 50)	Private Program (n = 98)	
Gender					0.120
Male	21.00 (63.60)	19.00 (48.70)	27.00 (54.00)	67.00 (68.40)	
Female	12.00 (36.40)	20.00 (51.30)	23.00 (46.00)	31.00 (31.60)	
Age (years)	64.27±12.11	66.38±11.74	68.04±10.71	67.18±11.81	0.417
BMI (kg/m^2^)	29.15±5.24	29.43±7.11	28.95±4.49	27.91±3.62	0.450
Ethnicity					0.026
White	18.00 (54.50)	30.00 (76.90)	29.00 (58.00)	70.00 (71.40)	
Brown-skinned	6.00 (18.20)	2.00 (5.10)	11.00 (22.00)	11.00 (11.20)	
Yellow	3.00 (9.10)	6.00 (15.40)	7.00 (14.00)	14.00 (14.30)	
Black^*^	6.00 (18.20)	1.00 (2.60)	3.00 (6.00)	3.00 (3.10)	
Treatment time (months)	54.48±56.98	28.33±22.20	74.34±65.79^A^	27.05 ± 20.57	0.002
Medication use	33.00 (100.00)	39.00 (100.00)	48.00 (96.00)	98.00 (100.00)	0.076
Occupation					0.225
Works	14.00 (42.40)	16.00 (41.00)	20.00 (40.00)	27.00 (27.60)	
Does not work	19.00 (57.60)	23.00 (59.00)	30.00 (60.00)	71.00 (72.40)	
City of residence					0.049
City X^*^	32.00 (97.00)	32.00 (82.10)	44.00 (88.00)	93.00 (94.90)	
Region	1.00 (3.00)	7.00 (17.90)	6.00 (12.00)	5.00 (5.10)	

Note: results are expressed in mean ± standard deviation or as a percentage and absolute number; Kruskal-Wallis test with Dunn’s post-test for quantitative variables or Chi-square test for categorical variables; *P*<0.05

A Value with a significant difference between low and high adherence in the private program

* Significant difference between categories

BMI=body mass index

**Table 2:** Characterization of patients concerning levels of anxiety and depression, mental status, socioeconomic level, level of education, and indication for CR according to the type of program and adherence.

Characterization	Low Adherence	Low Adherence	High Adherence	High Adherence	*P*-value
	Public Program (n = 33)	Private Program (n = 39)	Public Program (n = 50)	Private Program (n = 98)	
Anxiety, HADS	6.06±3.35^A^	3.43±3.68	4.84±3.72	3.71±3.30	0.001
Depression, HADS	4.27±3.23	3.79±3.32	4.18±3.63	2.85±2.37	0.056
MMSE	27.12±2.50	27.02±2.92	26.38±3.18	27.55±2.01	0.304
Socioeconomic level					< 0.001
A^*^	9.00 (27.30)	18.00 (46.20)	8.00 (16.00)	53.00 (54.10)	
B1	4.00 (12.10)	6.00 (15.40)	14.00 (28.00)	21.00 (21.40)	
B2	10.00 (30.30)	12.00 (30.80)	18.00 (36.00)	18.00 (18.40)	
C1^*^	7.00 (21.20)	2.00 (5.10)	8.00 (16.00)	3.00 (3.10)	
C2	3.00 (9.10)	1.00 (2.60)	2.00 (4.00)	3.00 (3.10)	
Level of education					0.195
Until complete high school	15.00 (45.50)	13.00 (33.30)	23.00 (46.00)	30.00 (30.60)	
Higher education and/or post-graduate	18.00 (54.50)	26.00 (66.70)	27.00 (54.00)	68.00 (69.40)	
Indication for CR					0.458
CAD	12.00 (36.40)	18.00 (46.20)	22.00 (44.00)	54.00 (55.10)	
MI	4.00 (12.10)	7.00 (17.90)	7.00 (14.00)	14.00 (14.30)	
Heart failure	1.00 (3.00)	1.00 (2.60)	3.00 (6.00)	0.00 (0.00)	
Rhythm disturbances	1.00 (3.00)	2.00 (5.10)	2.00 (4.00)	9.00 (9.20)	
Cardiomyopathy	5.00 (15.20)	4.00 (10.30)	4.00 (8.00)	4.00 (4.10)	
Others	5.00 (15.20)	3.00 (7.70)	4.00 (8.00)	8.00 (8.20)	
Risk factors	5.00 (15.20)	4.00 (10.30)	8.00 (16.00)	9.00 (9.20)	

Note: results are expressed in mean ± standard deviation or as a percentage and absolute number; “Others” mean Chagas disease, valvulopathy, aortic aneurysm, coronary anomaly, Marfan syndrome, constrictive pericarditis, and left ventricular hypertrophy; “Risk factors” are hypertension and diabetes; Kruskal-Wallis test with Dunn’s post-test for quantitative variables or Chi-square test for categorical variables; *P*<0.05

A Value with a significant difference between low and high adherence in the private program

* Significant difference between categories

CAD=coronary artery disease; CR=cardiac rehabilitation; HADS=Hospital Anxiety and Depression Scale; MI=myocardial infarction; MMSE=Mini-Mental State Examination

Tables 3 and 4 show adherence and barriers presented by questions and categories, also considering the level of adherence (low or high) and the characteristic of the program (public or private). Higher total barriers were found in PLA in public service compared to PHA in private service (*P*=0.023) - in the “perceived needs” subscale, PHA in the public service showed higher values when compared to PLA and PHA in the private sector (*P*<0.001) and the “access” barrier was higher for PHA in the public service compared to PHA in the private service (*P*=0.024).

**Table 3:** Adherence and barriers to cardiac rehabilitation according to the type of program and adherence.

Questionnaire	Low Adherence	Low Adherence	High Adherence	High Adherence	*P*-value
	Public Program (n = 33)	Private Program (n = 39)	Public Program (n = 50)	Private Program (n = 98)	
Adherence (percentage)	55.06±12.41 (58.33)	59.04±8.25 (61.11)	86.16±8.35 (87.49)^A,B^	85.06±7.36 (86.11)^A,B^	< 0.001
	[50.66 - 59.46]	[56.37 - 61.72]	[83.79 - 88.54]	[83.59 - 86.54]	
Total barriers	1.35±0.25	1.28±0.15	1.32±0.26	1.22±0.14	0.023
	(1.38)	(1.19)	(1.26)	(1.19)^A^	
	[1.26 - 1.44]	[1.23 - 1.33]	[1.25 - 1.39]	[1.19 - 1.25]	
Comorbidities/functional status	1.41±0.49	1.34±0.34	1.30±0.38	1.21±0.27	0.108
	(1.57)	(1.43)	(1.00)	(1.00)	
	[1.23 - 1.58)	[1.23- 1.45]	[1.19 - 1.41]	[1.15 - 1.26]	
Perceived need	1.11±0.26	1.06±0.20	1.19±0.36	1.02±0.13	< 0.001
	(1.00)	(1.00)	(1.00)^B,C^	(1.00)	
	[1.01 - 1.20]	[0.99 - 1.12]	[1.09 - 1.29]	[1.00 - 1.05]	
Personal/family issues	1.39±0.59	1.33±0.58	1.38±0.59	1.21±0.47	0.161
	(1.00)	(1.00)	(1.00)	(1.00)	
	[1.18 - 1.60]	[1.15 - 1.52]	[1.21 - 1.55]	[1.11 - 1.30]	
Work/time conflicts	2.35±1.54	2.02±1.17	2.14±1.25	2.16±1.27	0.838
	(1.50)	(1.00)	(2.25)	(2.50)	
	[1.80 - 2.89]	[1.65 - 2.40]	[1.78 - 2.50]	[1.91 - 2.42]	
Access	1.04±0.18	1.02±0.16	1.06±0.16	1.01±0.11	0.024
	(1.00)	(1.00)	(1.00)^C^	(1.00)	
	[0.98 - 1.11]	[0.97 - 1.08]	[1.01 - 1.11]	[0.99 - 1.04]	

Note: results are expressed in mean ± standard deviation (median) [lower limit - upper limit of the 95% confidence interval]; Kruskal-Wallis test with Dunn’s post-test, *P*<0.05

A Difference of low adherence, public program

B Difference of low adherence, private program

C Difference of high adherence, private program

**Table 4:** Questions about the Cardiac Rehabilitation Barriers Scale (CRBS).

I missed some sessions of CR because...	Low Adherence	Low Adherence	High Adherence	High Adherence	*P*-value
	Public Program (n = 33)	Private Program (n = 39)	Public Program (n = 50)	Private Program (n = 98)	
1. of distance	1.15±0.71	1.00±0.00	1.16±0.51	1.00±0.00	0.001
	(1.00)	(1.00)	(1.00)^A,C^	(1.00)	
	[0.89 - 1.40]	[1.00 - 1.00]	[0.89 - 1.40]	[1.00 - 1.00]	
2. of cost	1.03±0.17	1.10±0.64	1.08±0.27	1.00±0.00	0.048
	(1.00)	(1.00)	(1.00)	(1.00)^B^	
	[0.96 - 1.09]	[0.89 - 1.31]	[1.00 - 1.16]	[1.00 - 1.00]	
3. of transportation problems	1.12±0.54	1.10±0.64	1.38±1.05	1.11±0.64	0.005
	(1.00)	(1.00)	(1.00)^A,C^	(1.00)	
	[0.92 - 1.31]	[0.89 - 1.31]	[1.08 - 1.69]	[0.98 - 1.24]	
4. of family responsibilities	2.15±1.78	2.02±1.74	2.14±1.75	1.62±1.42	0.191
	(1.00)	(1.00)	(1.00)	(1.00)	
	[1.51 - 2.78]	[1.45 - 2.60]	[1.63 - 2.64]	[1.33 - 1.90]	
5. I didn’t know about CR	1.00±0.00	1.00±0.00	1.02±0.14	1.00±0.00	0.334
	(1.00)	(1.00)	(1.00)	(1.00)	
	[1.00 - 1.00]	[1.00 - 1.00]	[0.97 - 1.06]	[1.00 - 1.00]	
6. I don’t need CR	1.03±0.17	1.00±0.00	1.02±0.14	1.00±0.00	0.314
	(1.00)	(1.00)	(1.00)	(1.00)	
	[0.96 - 1.09]	[1.00 - 1.00]	[0.97 - 1.06]	[1.00 - 1.00]	
7. I already exercise at home or in my community	1.03±0.17	1.00±0.00	1.02±0.14	1.00±0.00	0.314
	(1.00)	(1.00)	(1.00)	(1.00)	
	[0.96 - 1.09]	[1.00 - 1.00]	[0.97 - 1.06]	[1.00 - 1.00]	
8. severe weather	1.60±1.95	1.36±1.10	1.61±1.41	1.14±0.70	0.092
	(1.00)	(1.00)	(1.00)	(1.00)	
	[0.91 - 2.29]	[1.00 - 1.73]	[1.20 - 2.01]	[1.00 - 1.28]	
9. I find exercise tiring or painful	1.15±0.71	1.00±0.00	1.04±0.28	1.00±0.00	0.060
	(1.00)	(1.00)	(1.00)	(1.00)	
	[0.89 - 1.40]	[1.00 - 1.00]	[0.95 - 1.12]	[1.00 - 1.00]	
10. travel	2.57±1.98	2.39±1.86	2.62±1.89	2.59±1.93	0.973
	(1.00)	(1.00)	(1.00)	(1.00)	
	[1.87 - 3.27]	[1.78 - 3.00]	[2.08 - 3.15]	[2.20 - 2.98]	
11. of time constraints	1.39±1.17	1.17±0.79	1.48±1.26	1.00±0.00	0.002
	(1.00)	(1.00)	(1.00)	(1.00)^B^	
	[0.97 - 1.80]	[0.92 - 1.43]	[1.12 - 1.83]	[1.00 - 1.00]	
12. of work responsibilities	2.12±1.79	1.5±1.40	1.66±1.47	1.73±1.55	0.399
	(1.00)	(1.00)	(1.00)	(1.00)	
	[1.48 - 2.75]	[1.13 - 2.04]	[1.23 - 2.08]	[1.42 - 2.04]	
13. I don’t have the energy	1.36±1.08	1.05±0.32	1.12±0.59	1.06±0.42	0.078
	(1.00)	(1.00)	(1.00)	(1.00)	
	[0.97 - 1.74]	[0.94 - 1.15]	[0.95 - 1.28]	[0.97 - 1.14]	
14. other health problems prevent me from going	2.69±2.00	3.02±2.00	2.38±1.90	2.22±1.81	0.134
	(1.00)	(1.00)	(1.00)	(1.00)	
	[1.98 - 3.40]	[2.37 - 3.67]	[1.83 - 2.92]	[1.85 - 2.58]	
15. I am too old	1.00±0.00	1.00±0.00	1.00±0.00	1.04±0.40	0.742
	(1.00)	(1.00)	(1.00)	(1.00)	
	[1.00 - 1.00]	[1.00 - 1.00]	[1.00 - 1.00]	[0.95 - 1.12]	
16. my doctor did not feel it was necessary	1.00±0.00	1.00±0.00	1.00±0.00	1.00±0.00	1.000
	(1.00)	(1.00)	(1.00)	(1.00)	
	[1.00 - 1.00]	[1.00 - 1.00]	[1.00 - 1.00]	[1.00 - 1.00]	
17. many people with heart problems don’t go, and they are fine	1.00±0.00	1.00±0.00	1.00±0.00	1.00±0.00	1.000
	(1.00)	(1.00)	(1.00)	(1.00)	
	[1.00 - 1.00]	[1.00 - 1.00]	[1.00 - 1.00]	[1.00 - 1.00]	
18. I can manage my heart problem on my own	1.00±0.00	1.00±0.00	1.00±0.00	1.00±0.00	1.000
	(1.00)	(1.00)	(1.00)	(1.00)	
	[1.00 - 1.00]	[1.00 - 1.00]	[1.00 - 1.00]	[1.00 - 1.00]	
19. I think I was referred, but the rehab program didn’t contact me	1.00±0.00	1.00±0.00	1.00±0.00	1.00±0.00	1.000
	(1.00)	(1.00)	(1.00)	(1.00)	
	[1.00 - 1.00]	[1.00 - 1.00]	[1.00 - 1.00]	[1.00 - 1.00]	
20. it took too long to get referred and into the program	1.00±0.00	1.00±0.00	1.00±0.00	1.06±0.42	0.475
	(1.00)	(1.00)	(1.00)	(1.00)	
	[1.00 - 1.00]	[1.00 - 1.00]	[1.00 - 1.00]	[0.97 - 1.14]	
21. I prefer to take care of my health alone, not in a group	1.03±0.17	1.00±0.00	1.00±0.00	1.00±0.00	0.131
	(1.00)	(1.00)	(1.00)	(1.00)	
	[0.96 - 1.09]	[1.00 - 1.00]	[1.00 - 1.00]	[1.00 - 1.00]	

Note: results are expressed in mean ± standard deviation (median) [lower limit - upper limit of the 95% confidence interval]; Kruskal-Wallis test with Dunn’s post-test, *P*<0.05

A Difference of low adherence, private program

B Difference of high adherence, public program

C Difference of high adherence, private program

CR=cardiac rehabilitation

The scale questions showed significant differences concerning distance (*P*=0.001) and problems with transportation (*P*=0.005), in which PHA in the public sector obtained higher barriers compared to PLA and PHA in the private sector, and about the cost (*P*=0.048) and time constraints (*P*=0.002), with PHA in the public sector presenting higher barriers than PHA in the private service.

## DISCUSSION

CR programs can have public and private funding, and studies indicate that there may be differences between these programs^[^4^,^5^,^8^]^, which can impact patients’ adherence to them. In this study, we began to investigate the differences in the characteristics of PLA and PHA to CR, both public and private, and whether there are differences in barriers to adherence between these patients and, eventually, some important differences appeared.

Regarding barriers, the results showed that PLA in the public program had higher “total barriers” when compared to PHA in the private program, moreover, PLA in the public service had the highest value of total barriers in the groups evaluated.

In the subscale “perceived need”, which includes a lack of knowledge and orientation about CR, PHA in the public service exhibited a higher barrier concerning PLA and PHA in the private service, and these same participants also presented a higher “access” barrier than PHA in the private service. Domain related to logistical factors can influence adherence whereas the members attend the sessions more when the place is more accessible and does not require long distance traveling^[^2^,^4^,^15^]^. These results are similar to other studies that demonstrated that the main barriers in the public program are “perceived need” and “access”^[^2^,^4^,^6^,^15^]^.

Concerning the CRBS questions that consist of the “perceived need” subscale, “of time constraints” and “transportation problems” were the most common in PHA in the public program. Public transportation in the city of X presents some problems such as lack of vehicles, long routes, and long waiting times between buses^[^16^]^ and as many patients depend on this type of transport or other people to get to the program location, this may be related to the “transportation problems” question. Also, part of the participants works regularly, which may have influenced the “of time constraints” question. These results contradict other studies^[^2^,^4^,^17^]^ that pointed out the lack of orientation and knowledge about the beneficial effects of CR as the highest barriers within this subscale. The divergence observed may be related to educational classes that occur monthly and with high participation in the evaluated programs, which minimize these barriers^[^18^]^.

Even the questions about “distance” and “cost” that compose the subscale “access” were also higher for the PHA of the public service. These questions are related to the distance between the home or city where the patients live, the need to spend on the program, for example, with public transportation or fuel^[^17^]^, and displacement over a long period^[^19^]^, however, they were not able to affect the adherence of these individuals.

As expected, PHA in both public and private services showed higher adherence compared to PLA in the public and private services. As for clinical and sociodemographic characteristics, inequality in the participation of black individuals was evidenced^[^15^]^, having a low participation rate with the higher number of individuals present in the public service and the PLA group. Concerning treatment time, patients in the public program have longer treatment time compared to patients in the private program, with statistical significance for the PHA in the public program compared to the two groups in the private program, which may be, at least in part, related to the absence of monthly payment and the non-dependence on health plans to release sessions in the public service.

The evaluated patients have a low level of anxiety, which can be connected to the practice of exercise, which generates benefits in physical, mental, and social functioning^[^1^-^3^]^ and relieves anxiety symptoms^[^20^]^. However, anxiety levels were higher in PLA in the public service when compared to PLA and PHA in the private program. Significant differences between groups were found for socioeconomic levels. Class C has a greater number of patients in the public service, while class A have a greater number of patients in the private service, indicating greater socioeconomic power of these participants. The level of education, which generally depends on the socioeconomic level, indicates that the higher the level of education, the lower the barriers, and the higher the level of adherence to CR^[^5^,^10^]^. Our results corroborate these aspects because most patients in the private service have a higher level of education, a higher socioeconomic level, and lower barriers.

As general characteristics of the groups, there was a lower prevalence of women, as already demonstrated in another study^[^8^]^. Individuals with an average age > 60 years predominated, which reinforces the importance of encouraging this population to participate and have good adherence to CR, considering the benefits and low risk presented in this modality^[^21^]^. Still, the average BMI of all groups > 27.91 kg/m^2^, individuals who do not work, use medication, have completed higher education and/or postgraduation, with a low level of depression, good cognitive status, and diagnosis of coronary artery disease as the main indication for participation in the programs prevail. Also, most individuals reside in the same city as the CR, but there are more PLA in the private program who reside in the region, which can interfere with these individuals’ adherence.

It is important to highlight that the barriers to adherence to treatment involve not only the patient but also the professional and the health system^[^22^]^, requiring strategies in all these areas for better results.

In this context, some strategies have been proposed to improve adherence to CR, such as home rehabilitation^[^2^]^, unsupervised modalities, like using apps on mobile^[^23^]^, use of virtual reality in CR^[^24^]^, use of cognitive and behavioral elements, training for changes in lifestyle, presence of a doctor in the program area, and adequate space and equipment^[^25^]^. Besides, programs must be broad, simple, and low cost, these strategies being able to minimize the barriers found in this study.

### Limitations

As limitations of the study, we point out the loss of participants due to logistical problems, as well as a smaller number of participants with low adherence and belonging to the public program, which may have interfered with the results. Furthermore, individuals were recruited from a specific region of Brazil and from a public and a private program, which may not represent the reality of the entire country, due to cultural, socioeconomic, and programs offering differences. However, the studies in the literature analyze only the barriers and do not consider the level of adherence of the participants, highlighting the importance of considering this aspect since the participants present different barriers according to the level of adherence.

## CONCLUSION

Finally, we conclude that the main barriers observed in the analyzed programs were “total barriers”, “perceived need”, and “access”, mainly questions related to cost, distance, transportation problems, and time constraints, being the patients with high adherence in the public program those who present more barriers.

Still, some general characteristics when evaluating the type of program and the level of adherence show differences regarding ethnicity, socioeconomic and anxiety levels, city of residence, and treatment time.
